# Age‐dependent effects of thoracic and capillary blood volume distribution on pulmonary artery pressure and lung diffusing capacity

**DOI:** 10.14814/phy2.13834

**Published:** 2018-09-02

**Authors:** Kirsten E. Coffman, Matthew G. Boeker, Alex R. Carlson, Bruce D. Johnson

**Affiliations:** ^1^ Department of Cardiovascular Diseases Mayo Clinic Rochester Minnesota

**Keywords:** Aging, cardiac output, pulmonary smooth muscle tone, pulmonary vascular distensibility, pulmonary vasculature

## Abstract

Aging is associated with pulmonary vascular remodeling and reduced distensibility. We investigated the influence of aging on changes in cardiac output (Q), mean pulmonary artery pressure (mPAP), and lung diffusing capacity in response to alterations in thoracic blood volume. The role of pulmonary smooth muscle tone was also interrogated via pulmonary vasodilation. Nine younger (27 ± 4 years) and nine older (71 ± 4 years) healthy adults reached steady‐state in a Supine (0°), Upright (+20°), or Head‐down (−20°) position in order to alter thoracic blood volume. In each position, echocardiography was performed to calculate mPAP and Q, and lung diffusing capacity for carbon monoxide (DLCO) and nitric oxide (DLNO) was assessed. Next, 100 mg sildenafil was administered to reduce pulmonary smooth muscle tone, after which the protocol was repeated. mPAP (*P* ≤ 0.029) and Q (*P* ≤ 0.032) were lower in the Upright versus Supine and Head‐down positions, and mPAP was reduced following sildenafil administration (*P* = 0.019), in older adults only. SV was lower in the Upright versus Supine and Head‐down positions in both younger (*P* ≤ 0.008) and older (*P* ≤ 0.003) adults. DLCO and DLNO were not greatly altered by position changes or sildenafil administration. However, the DLNO/DLCO ratio was lower in the Supine and/or Head‐down positions (*P* ≤ 0.05), but higher following sildenafil administration (*P* ≤ 0.007), in both younger and older adults. In conclusion, older adults experience greater cardiopulmonary alterations following thoracic blood volume changes, and pulmonary smooth muscle tone plays a role in resting mPAP in older adults only. Furthermore, mPAP is an important determinant of pulmonary capillary blood volume distribution (DLNO/DLCO), regardless of age.

## Introduction

Aging is associated with remodeling of the pulmonary artery and vein (Fernie and Lamb 1986; Harris et al. 1965; Hosoda et al. 1984; Mackay et al. 1978; Plank et al. 1980; Warnock and Kunzmann 1977) and a reduction in pulmonary vascular distensibility as assessed by ex vivo methods (Banks et al. 1978; Gozna et al. 1974; Harris et al. 1965; Mackay et al. 1978; Reeves et al. 2005). However, measures of pulmonary vascular distensibility are challenging to assess in vivo, and while a modeling approach has previously been utilized it requires assessment of multiple exercise stages which is not always feasible (Lalande et al. 2012; Linehan et al. 1992; Reeves et al. 2005). Thus, we sought to use a simple, noninvasive method at rest in order to assess age‐dependent differences in variables that are related to pulmonary vascular distensibility. Specifically, we used passive changes in body position to investigate the age‐dependent responses of mean pulmonary artery pressure (mPAP) and cardiac output (Q) in healthy individuals.

Remodeling of the pulmonary vasculature is likely in part responsible for any age‐dependent difference in the mPAP response to positional changes; however, alterations in pulmonary smooth muscle tone may also play a role. It has previously been shown that pulmonary endothelial function is reduced in older adults (Jane‐Wit and Chun 2012; Seals and Esler 2000). Additionally, muscle sympathetic nerve activity (MSNA), at least systemically (i.e., skeletal muscle), is also increased in older adults (Jane‐Wit and Chun 2012; Seals and Esler 2000). Thus, reduced endothelial function, and potentially increased MSNA activity within the pulmonary vasculature (i.e., pulmonary vascular smooth muscle), may increase pulmonary smooth muscle tone in older adults and contribute to differences in the mPAP response to positional changes.

Healthy aging is also associated with a reduction in lung diffusing capacity, or the ability to transfer gases from the pulmonary alveoli to capillaries (Chang et al. 1992; Guenard and Marthan 1996; Stam et al. 1994). Lung diffusing capacity provides a functional representation of the lung surface area available for gas exchange and is a standard aspect of clinical pulmonary function testing. It is well established that the reduction in lung diffusing capacity in older adults is partially an outcome of a reduction in the alveolar‐capillary surface area available for gas exchange, caused by both a decrease in number of pulmonary alveoli and thus an increase in pulmonary alveolar size as well as a decrease in the density of pulmonary capillaries (Butler and Kleinerman 1970; Gillooly and Lamb 1993; Thurlbeck and Angus 1975; Verbeken et al. 1992). However, differences in mPAP may also alter the distribution of pulmonary capillary blood volume throughout the vascular network, and thus have an effect on surface area for gas exchange and subsequently lung diffusing capacity.

The present study was an investigation into the pulmonary vascular response to variations in thoracic blood volume following passive changes in body position in younger versus older adults. First, we aimed to investigate whether older individuals experienced a differential effect of body position on mPAP and Q versus younger adults. Second, we aimed to understand the role of pulmonary smooth muscle tone, as opposed to vascular remodeling, on measures of mPAP in older versus younger adults. Third, we aimed to interrogate the effect of mPAP on lung diffusing capacity in healthy individuals. We hypothesized that older individuals would experience an increase in mPAP following an increase in thoracic fluid volume, that smooth muscle tone would play a larger role in mPAP in older versus younger adults, and that variations in mPAP would alter lung diffusing capacity in both younger and older individuals.

## Methods

### Subjects

Nine younger (5M/4F, 27 ± 4 years) and nine older (4M/5F, 71 ± 4 years) nonsmoking adults with no history of respiratory, cardiovascular, or metabolic diseases participated in the study (Table 1). Participants were recruited from the general population and were moderately active individuals. Participants were not taking any cardiac, pulmonary, or metabolic medications, nor any other medications that could potentially alter the physiological parameters of interest. Each participant gave written informed consent after being provided a detailed description of the study requirements. The experimental procedures were approved by the Mayo Clinic Institutional Review Board and were performed in accordance with the ethical standards of the Declaration of Helsinki.

**Table 1:** Subject demographics

	Younger	Older	*P* ‐Value
Subjects, N (M/F)	9 (5/4)	9 (4/5)	
Age, years	27.0 ± 3.6	71.3 ± 3.6	<0.001
Height, cm	179 ± 8	170 ± 7	0.027
Weight, kg	75.4 ± 8.4	70.0 ± 10.8	0.255
BMI, kg/m^2^	23.6 ± 2.2	24.2 ± 2.7	0.666
BSA, m^2^	1.93 ± 0.14	1.81 ± 0.17	0.124
FVC, % pred.	100 ± 13	103 ± 18	0.742
FEV_1_, % pred.	100 ± 12	105 ± 18	0.440
FEV_1_/FVC, % pred.	99 ± 6	102 ± 7	0.340

BMI, body mass index; BSA, body surface area; FVC, forced vital capacity; FEV_1_, forced expiratory volume in 1 sec.

### Study overview

The experimental procedures were completed during two separate visits to the laboratory. At visit 1, pulmonary function was assessed according to standard procedure (Miller et al. 2005); subjects were excluded if their pulmonary function (FVC, FEV_1_, and PEF) was below 80% of predicted (Hankinson et al. 1999). Next, subjects underwent an echocardiographic screening by a trained sonographer and were excluded if an adequate tricuspid regurgitation (TR) could not be visualized and quantified.

At visit 2, subjects lied flat on a tilt table for 10 min in order to reach hemodynamic steady‐state conditions in three positions: Supine (0°), Upright (+20°), or Head‐down (−20°). Altering body position during echocardiography measurements has been successfully utilized in younger individuals to investigate changes in various aspects of cardiac function (Rowland et al. 2012). Echocardiography and duplicate measures of lung diffusing capacity were performed in each position. Next, 100 mg sildenafil, a PDE5 inhibitor, was administered orally followed by a 60 min rest period in order for sildenafil to take effect. During this rest period, subjects remained in a semi‐supine position and were allowed water ad libitum. Then, the protocol was repeated.

### Echocardiography

The same trained sonographer performed all echocardiographic measurements throughout the study. At the beginning of the protocol in the supine position, right atrial pressure (RAP) was estimated via the inferior vena cava diameter and percent collapse during a sniff maneuver (Kircher et al. 1990) and left ventricular outflow tract diameter (D_LVOT_) was also measured. These values of RAP and D_LVOT_ were used for all subsequent calculations. Maximum tricuspid regurgitation velocity (TRVmax) and time velocity integral of the left ventricular outflow tract (TVI_LVOT_) were collected in each position. Each of these measures was collected in the sonographic window determined optimal by the sonographer and remained consistent throughout positions for each subject. Additionally, all values were determined via an average of 3–5 images. These measures, in addition to HR via 3‐lead ECG, were used to calculate systolic pulmonary artery pressure (sPAP) and thus mean pulmonary artery pressure (mPAP), stroke volume (SV), and cardiac output (Q), as per Yock and Popp (1984), Chemla et al. (2004) and Huntsman et al. (1983):

$$\text{sPAP}=4\times {\text{TRV}}_{\max}^{2}+\text{RAP}$$

mPAP=(0.6×sPAP)+2

$$\text{SV}=3.14\times {\left(D_{\frac{\mathrm{LVOT}}{2}}\right)}^{2}\times {\text{TVI}}_{\mathrm{LVOT}}$$

Q=SV×HR

### Lung diffusing capacity via rebreathe

Lung diffusing capacity for carbon monoxide (DLCO) and lung diffusing capacity for nitric oxide (DLNO) were assessed using a rebreathe technique by taking advantage of the diffusion‐limited nature of CO and NO gas (Hsia et al. 1995; Tamhane et al. 2001). Briefly, DLCO and DLNO were determined via the rate of disappearance of CO and NO, respectively. Following a normal expiration, subjects were switched into a rebreathe bag containing the test gas mixture (0.3% C^18^O, 45 ppm NO, 9% He, 0.6% C_2_H_2_, 35% O_2_ and N_2_ balance) and instructed to nearly empty the bag with each breath for 8‐10 consecutive breaths. This maneuver was performed in duplicate (once before and once after echocardiographic measurements) in each position.

### Statistical analyses

Younger versus older demographics (Table 1) were compared using an independent samples *t*‐test. The effect of position within each age cohort was analyzed using repeated measures ANOVA with Tukey post hoc comparisons following significant main effects. The effect of sildenafil administration on Supine values within an age cohort were compared using a paired samples *t*‐test. Statistical analyses were performed in GraphPad Prism 7.02 (GraphPad Software, Inc., La Jolla, CA) with statistical significance set at *P* < 0.05.

## Results

### Subjects

Subject characteristics and pulmonary function are shown in Table 1. While the younger cohort was significantly taller versus the older cohort (179 ± 8 vs. 170 ± 7 cm, *P* = 0.027), the younger and older cohorts were well‐matched for weight as well as BMI and BSA. All subjects had pulmonary function within normal limits and percent‐predicted values were not different between younger and older individuals.

### Mean pulmonary artery pressure

**Figure 1:**
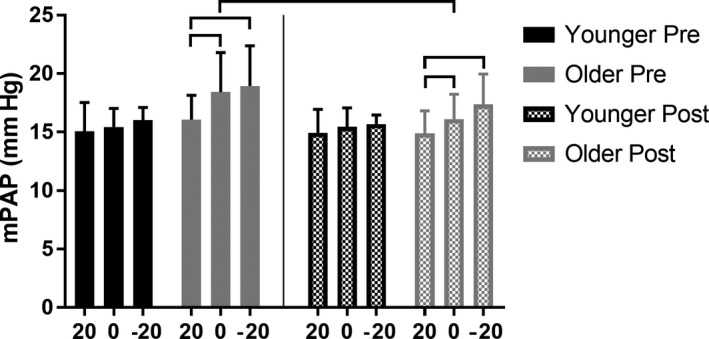
The effect of passive body position changes on mean pulmonary artery pressure (mPAP) in younger (<35 years) and older (>65 years) individuals before (“Pre”) and after (“Post”) sildenafil administration. 20, Upright; 0, Supine; −20, Head‐down. Horizontal bars identify significance at *P *<* *0.05. mPAP was lower in the Upright versus Supine and Head‐down positions, both pre‐ and post‐sildenafil, in older adults only. mPAP decreased following sildenafil administration in older adults only.

In younger adults, there was no effect of position on mPAP, either pre‐ or post‐sildenafil. Additionally, there was no change in Supine values of mPAP following sildenafil administration (Fig. 1; pre 15.4 ± 1.6 vs. post 15.5 ±1.6 mmHg, *P* = 0.327; Δ_sildenafil_ = 0.03 ± 0.7 mmHg). However, in older adults mPAP was lower in the Upright versus Supine and Head‐down positions both pre‐sildenafil (Fig. 1; Upright 16.1 ± 2.1 vs. Supine 18.4 ± 3.4 mmHg, *P* = 0.022 and Head‐down 18.9 ± 3.4 mmHg, *P* = 0.029) and post‐sildenafil (Fig. 1; Upright 14.9 ± 1.9 vs. Supine 16.1 ± 2.1 mmHg, *P* = 0.013 and Head‐down 17.4 ± 2.6 mmHg, *P* = 0.007). Additionally, mPAP was decreased in the Supine position following sildenafil administration (Fig. 1, pre 18.4 ± 3.4 versus post 16.1 ± 2.1 mmHg, *P* = 0.019; Δ_sildenafil_ = −2.3 ± 2.2 mmHg). These data demonstrate that a passive reduction in thoracic blood volume, as well as a reduction in pulmonary smooth muscle tone, both decrease mean pulmonary artery pressure in older individuals only.

### Cardiac output, stroke volume, and heart rate

**Figure 2:**
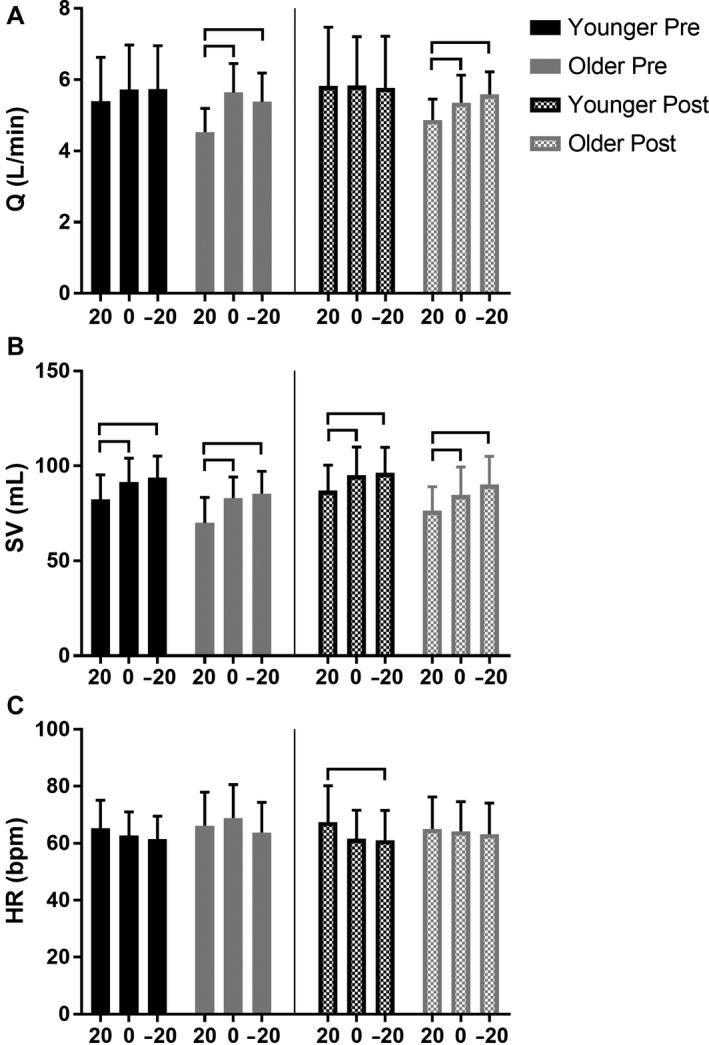
The effect of passive body position changes on cardiac output (Q), stroke volume (SV), and heart rate (HR) in younger (<35 years) and older (>65 years) individuals before (“Pre”) and after (“Post”) sildenafil administration. 20, Upright; 0, Supine; −20, Head‐down. Horizontal bars identify significance at *P *<* *0.05. Q was lower in the Upright versus Supine and Head‐down positions, both pre‐ and post‐sildenafil, in older adults only. SV was lower in the Upright versus Supine and Head‐down positions, both pre‐ and post‐sildenafil, in younger and older adults.

In younger adults, there was no effect of position on Q, either pre‐ or post‐sildenafil (Fig. 2A). However, SV was lower in the Upright versus Supine and Head‐down positions both pre‐sildenafil (Fig. 2B; Upright 82.5 ± 12.9 vs. Supine 91.5 ± 12.6 mL, *P* = 0.001 and Head‐down 93.8 ± 11.4 mL, *P* = 0.008) and post‐sildenafil (Fig. 2B; Upright 87.1 ± 13.4 vs. Supine 95.1 ± 14.9 mL, *P* = 0.001 and Head‐down 96.3 ± 13.5 mL, *P* = 0.007) in younger adults. In the post‐sildenafil condition, HR was lower in the Upright versus Head‐down position (Fig. 2B; Upright 67.4 ± 12.7 vs. Head‐down 61.1 ± 10.4 bpm, *P* = 0.044) in younger individuals.

Alternatively, in older adults Q was lower in the Upright vs. Supine and Head‐down positions both pre‐sildenafil (Fig. 2A; Upright 4.5 ± 0.7 vs. Supine 5.6 ± 0.8 L*min^−1^, *P* = 0.003 and Head‐down 5.4 ± 0.8 L*min^−1^, *P* = 0.001) and post‐sildenafil (Fig. 2A; Upright 4.9 ± 0.6 vs. Supine 5.4 ± 0.8 L*min^−1^, *P* = 0.032 and Head‐down 5.6 ± 0.6 L*min^−1^, *P* < 0.001). SV was also lower in the Upright vs. Supine and Head‐down positions both pre‐sildenafil (Fig. 2B; Upright 70.0 ± 13.4 vs. 83.0 ± 11.1 mL, *P* = 0.003 and Head‐down 85.4 ± 11.8 mL, *P* < 0.001) and post‐sildenafil (Fig. 2B; Upright 76.4 ± 12.6 vs. Supine 84.7 ± 14.7 mL, *P* = 0.002 and Head‐down 90.2 ± 14.9 mL, *P* < 0.001) in older individuals. There was no effect of position on HR in older individuals (Fig. 2C). Additionally, there was no change in Supine values of Q, SV, or HR following sildenafil administration in either younger or older individuals (Fig. 2).

All in all, these data suggest that a passive decrease in thoracic blood volume elicits a reduction in SV, regardless of age. However, Q is not altered in younger adults, possibly due to a corresponding increase in HR not experienced by older adults in the Upright position.

### Lung diffusing capacity

**Figure 3:**
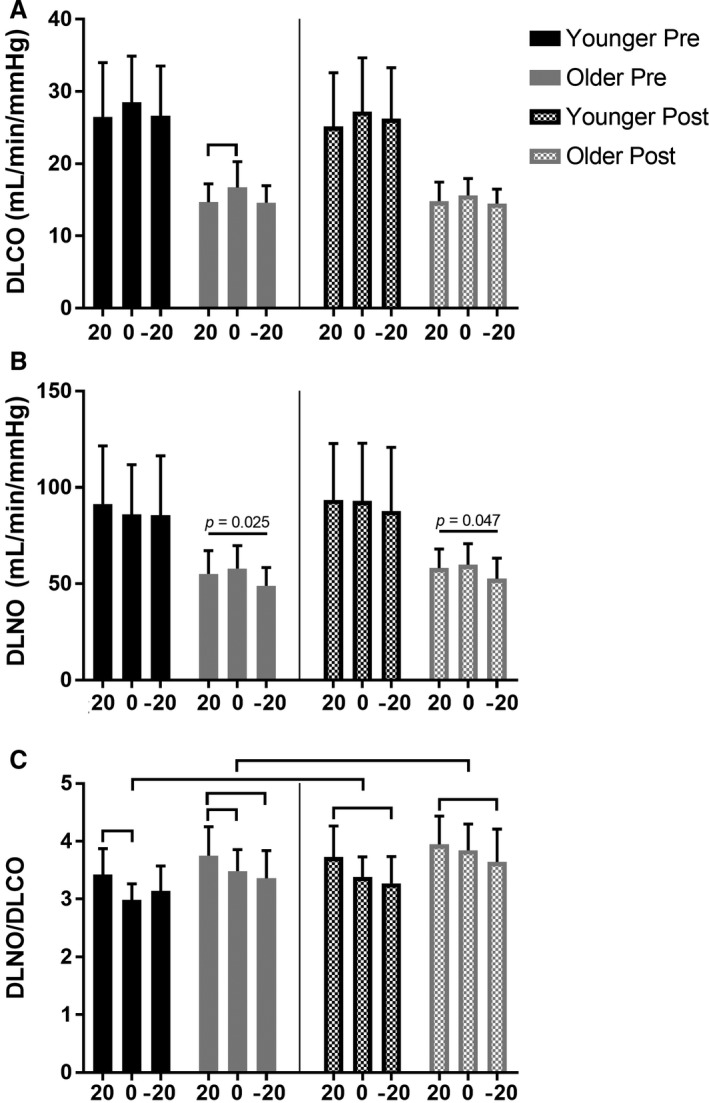
The effect of passive body position changes on lung diffusing capacity for carbon monoxide (DLCO), nitric oxide (DLNO), and the DLNO/DLCO ratio in younger (<35 years) and older (>65 years) individuals before (“Pre”) and after (“Post”) sildenafil administration. 20, Upright; 0, Supine; −20, Head‐down. Horizontal bars identify significance at *P *<* *0.05. DLNO/DLCO was lower in the Upright versus Supine and/or Head‐down positions, both pre‐ and post‐sildenafil, in younger and older adults. DLNO/DLCO increased following sildenafil administration in both younger and older adults.

In younger adults, there was no effect of position or sildenafil administration on DLCO or DLNO (Fig. 3A–B). In older adults, DLCO was lower in the Upright versus Supine position pre‐sildenafil only (Fig. 3A; Upright 14.7 ± 2.5 vs. Supine 16.7 ± 3.6 mL*min^−1^*mmHg^−1^, *P* = 0.030). Additionally, in older adults there was a significant main effect of position on DLNO both pre‐sildenafil (Fig. 3B; *P* = 0.025) and post‐sildenafil (Fig. 3B; *P* = 0.047); however, post hoc comparisons did not yield any significant differences between any two positions. Additionally, there was no effect of sildenafil administration on Supine values of DLCO and DLNO in younger or older adults (Fig. 3A–B).

Importantly, the DLNO/DLCO ratio can also be calculated and used to distinguish the relative importance of pulmonary capillary blood volume on lung diffusing capacity, where a decrease in DLNO/DLCO suggests an increased role of pulmonary capillary blood volume (Glenet et al. 2007). In general, DLNO/DLCO was higher in the Upright vs. Supine and/or Head‐down positions, both pre‐ and post‐sildenafil, in both younger and older individuals (Fig. 3C; all horizontal bars signify *P* < 0.05). Furthermore, in both younger and older adults, DLNO/DLCO was increased in the Supine position following sildenafil administration (Fig. 3C, both *P* ≤ 0.007). These data suggest that changes in thoracic blood volume and pulmonary smooth muscle tone alter pulmonary capillary blood volume distribution in both younger and older adults.

## Discussion

### Major findings

The aim of this study was to investigate the pulmonary vascular response to changes in thoracic blood volume in younger versus older adults. The main findings were as follows: (1) only older individuals experienced a reduction in mPAP and Q in the Upright versus Supine and Head‐down positions, (2) a reduction in pulmonary smooth muscle tone via sildenafil decreased Supine values of mPAP in older individuals only, and (3) in both younger and older individuals, an increase in thoracic blood volume reduced DLNO/DLCO, while a reduction in pulmonary smooth muscle tone increased DLNO/DLCO. These data suggest that older individuals are less able to maintain cardiopulmonary homeostasis following passive changes in thoracic blood volume. These data also demonstrate the greater importance of pulmonary smooth muscle tone on mPAP in older versus younger individuals. Furthermore, even at rest, mPAP plays a role in pulmonary capillary blood volume distribution in both younger and older healthy adults.

### Increase in mPAP and Q in older adults

Presently, we observed a reduction in mPAP following a reduction in thoracic blood volume in the Upright versus Supine and Head‐down positions in older individuals only. This is in contrast to our hypothesis that, due to an age‐dependent decrease in pulmonary vascular distensibility, an increase in thoracic blood volume would elicit a greater increase in mPAP in older versus younger adults. It is well‐established that Q and mPAP are intimately linked (Kovacs et al. 2009; Reeves et al. 2005); thus, it is possible that the reduction in mPAP is simply an outcome of the concomitant reduction in Q observed in older individuals in the Upright position. This reduction in Q in the Upright position may be due to a reduction in venous return and thus SV. However, younger individuals also experienced a decrease in SV but with no reduction in Q in the Upright position. In the post‐sildenafil condition, younger individuals did experience a corresponding increase in HR in the Upright position, which could explain the lack of a reduction in Q. However, there was no increase in HR in the Upright position before sildenafil administration, so this explanation does not fully elucidate the age‐dependent difference in the Q response to positional changes.

Alternatively, it is possible that the greater mPAP in older individuals in the Supine and Head‐down positions may be evidence of a decreased pulmonary vascular distensibility and thus increased pulmonary vascular resistance (PVR). It is well‐established that PVR is higher in older adults during exercise (Ehrsam et al. 1983; Emirgil et al. 1967; van Empel et al. 2014), a stressor that increases thoracic blood volume. Thus, perhaps in older adults a larger thoracic blood volume in the Supine and Head‐down positions interacts with an age‐dependent decrease in pulmonary vascular distensibility, resulting in increased PVR and therefore mPAP. However, in the Upright position with a lower thoracic blood volume, mPAP values in older adults may be able to return to the lower values seen in younger adults. Nevertheless, this is speculative, and whether this potential effect of decreased pulmonary vascular distensibility is driven by vascular wall remodeling and/or increased pulmonary smooth muscle tone is unclear.

### Role of pulmonary smooth muscle tone on mPAP

The effect of resting pulmonary smooth muscle tone on mPAP is not established in healthy individuals. Presently, sildenafil administration yielded a reduction in mPAP in older adults only. These results might suggest that older individuals experience a greater resting pulmonary smooth muscle tone, such that administration of sildenafil, a PDE5 inhibitor, elicits substantial vasodilation and a concomitant reduction in mPAP. It is possible that this increase in tone may be an outcome of a reduction in endothelial function observed in older adults (Jane‐Wit and Chun 2012; Muller et al. 2002; Seals et al. 2009). Theoretically, it is also possible that an age‐dependent increase in mPAP may exist to combat pulmonary vascular remodeling. In other words, a greater driving pressure into the pulmonary vasculature may be required to maintain adequate pulmonary capillary recruitment and thus sufficient lung surface area for gas exchange (and DLCO) in older adults.

### Role of mPAP on lung diffusing capacity

DLCO and DLNO are reduced in older individuals (Chang et al. 1992; Coffman et al. 2017; Guenard and Marthan 1996; Stam et al. 1994) due primarily to a reduction in the alveolar‐capillary surface area available for gas exchange (Butler and Kleinerman 1970; Gillooly and Lamb 1993; Thurlbeck and Angus 1975; Verbeken et al. 1992). However, the importance of mPAP on pulmonary capillary blood volume distribution and thus DLCO and DLNO is not known. Modeling of DLNO/DLCO suggests that a fall in this ratio is indicative of a thickening of the pulmonary capillary blood sheet (Glenet et al. 2007). In other words, a fall in DLNO/DLCO suggests that measures of lung diffusing capacity have become relatively more reliant on the capillary blood volume component of gas transfer from the pulmonary alveoli to hemoglobin. Presently, an increase in thoracic blood volume from the Upright to Supine to Head‐down position caused decreases in DLNO/DLCO in both younger and older individuals. This reduction in DLNO/DLCO following passive increases in thoracic blood volume suggests an increased diffusion distance from the pulmonary alveoli to hemoglobin, likely indicative of pulmonary capillary engorgement and distension. Pulmonary capillary distension might be anticipated given a passive increase in thoracic blood volume from the Upright to Supine to Head‐down position.

More notably, DLNO/DLCO increased following a reduction in pulmonary smooth muscle tone in both younger and older adults, indicative of a reduction in the pulmonary capillary blood volume component of lung diffusing capacity (Glenet et al. 2007). Theoretically, a reduction in smooth muscle tone may cause a de‐recruitment of pulmonary capillaries, thus decreasing the lung surface area of alveoli in contact with blood. In other words, mPAP, the driving pressure into the pulmonary vascular network, may become inadequate to maintain an ideal match between perfusion and ventilation, thus resulting in a lower lung surface area available for gas exchange. At rest, this effect was not substantial enough to significantly reduce DLCO (or DLNO) following sildenafil administration; however, we have recently shown that, during exercise, the impact of mPAP on DLCO is significant (Coffman et al. 2018). All in all, these data highlight the potential influence of mPAP on pulmonary capillary blood volume distribution.

### Impact of sildenafil on systemic circulation

Sildenafil is a PDE‐5 inhibitor that, in addition to acting on the pulmonary circulation, also acts throughout the systemic circulation. Clinically, pulmonary hypertension patients are prescribed 20 mg of sildenafil three times per day. Thus, this study used a relatively large dose of sildenafil (100 mg) that led to a modest decrease in mean systemic arterial pressure in both younger (pre 79 ± 5 vs. post 77 ± 7 mmHg, *P* = 0.044) and older (pre 77 ± 9 vs. post 73 ± 8 mmHg, *P* = 0.005) individuals. Because the pulmonary and systemic circulations are intimately linked, this decrease in systemic blood pressure could theoretically be responsible for some of the pulmonary effects of sildenafil observed presently. However, there were no changes in supine steady state heart rate, stroke volume, or cardiac output (Fig. 2), suggesting that the systemic effects of sildenafil on the results presented here are minimal.

### Clinical implications

In this study, a pharmacological reduction in mPAP yielded an increase in DLNO/DLCO regardless of age, suggesting a reduction in the lung surface area of alveoli in contact with blood. However, in clinical conditions such as pulmonary hypertension where mPAP is increased, sildenafil has been shown to improve lung diffusing capacity (Vitulo et al. 2017). Thus, we have previously hypothesized a “sweet spot” for pulmonary pressures, in which a reduction in the driving pressure into the pulmonary circulation (mPAP) may negatively impact ventilation‐perfusion matching, while an increase in pulmonary pressures can also be deleterious in clinical conditions (Coffman et al. 2018). Therefore, the present findings demonstrate a potentially delicate balance between pulmonary pressures and lung diffusing capacity.

## Conclusions

In conclusion, only older individuals experience a lower mPAP and Q with a reduction in thoracic blood volume, potentially indicative of differences in HR response or decreased pulmonary vascular distensibility with aging. Furthermore, sildenafil reduced mPAP in older adults only, suggesting an increase in pulmonary smooth muscle tone at rest with aging. Importantly, pulmonary capillary blood volume distribution, a determinant of lung diffusing capacity, is likely altered by changes in mPAP in both younger and older adults, even at rest.

## Conflict of interest

None declared.
